# Prevention of Early Alzheimer’s Disease by Erinacine A-Enriched *Hericium erinaceus* Mycelia Pilot Double-Blind Placebo-Controlled Study

**DOI:** 10.3389/fnagi.2020.00155

**Published:** 2020-06-03

**Authors:** I-Chen Li, Han-Hsin Chang, Chuan-Han Lin, Wan-Ping Chen, Tsung-Han Lu, Li-Ya Lee, Yu-Wen Chen, Yen-Po Chen, Chin-Chu Chen, David Pei-Cheng Lin

**Affiliations:** ^1^Biotech Research Institute, Grape King Bio Ltd., Taoyuan City, Taiwan; ^2^Department of Nutrition, Chung Shan Medical University, Taichung City, Taiwan; ^3^Department of Medical Laboratory and Biotechnology, Chung Shan Medical University, Taichung City, Taiwan; ^4^Institute of Food Science and Technology, National Taiwan University, Taipei City, Taiwan; ^5^Department of Food Science, Nutrition and Nutraceutical Biotechnology, Shih Chien University, Taipei City, Taiwan; ^6^Department of Bioscience Technology, Chung Yuan Christian University, Taoyuan City, Taiwan; ^7^Department of Ophthalmology, Chung Shan Medical University Hospital, Taichung City, Taiwan

**Keywords:** erinacine A-enriched *H. erinaceus* mycelia, Alzheimer’s disease, pilot study, prevention, magnetic resonance imaging

## Abstract

**Objective:**

To investigate the efficacy and safety of three *H. erinaceus* mycelia (EAHE) capsules (350 mg/capsule; containing 5 mg/g erinacine A active ingredient) per day for the treatment of patients with mild Alzheimer’s Disease (AD).

**Methods:**

This study comprised a 3-week no-drug screening period, followed by a 49-week double-blind treatment period with 2-parallel groups in which eligible patients were randomized to either three 5 mg/g EAHE mycelia capsules per day or identical appearing placebo capsules. Cognitive assessments, ophthalmic examinations, biomarker collection, and neuroimaging were followed throughout the study period.

**Results:**

After 49 weeks of EAHE intervention, a significant decrease in Cognitive Abilities Screening Instrument score was noted in the placebo group, a significant improvement in Mini-Mental State Examination score was observed in the EAHE group and a significant Instrumental Activities of Daily Living score difference were found between the two groups. In addition, EAHE group achieved a significantly better contrast sensitivity when compared to the placebo group. Moreover, only the placebo group observed significantly lowered biomarkers such as calcium, albumin, apolipoprotein E4, hemoglobin, and brain-derived neurotrophic factor and significantly elevated alpha1-antichymotrypsin and amyloid-beta peptide 1–40 over the study period. Using diffusion tensor imaging, the mean apparent diffusion coefficient (ADC) values from the arcuate fasciculus region in the dominant hemisphere significantly increased in the placebo group while no significant difference was found in the EAHE group in comparison to their baselines. Moreover, ADC values from the parahippocampal cingulum region in the dominant hemisphere significantly decreased in the EAHE group whereas no significant difference was found in the placebo group when compared to their baselines. Lastly, except for four subjects who dropped out of the study due to abdominal discomfort, nausea, and skin rash, no other adverse events were reported.

**Conclusion:**

Three 350 mg/g EAHE capsules intervention for 49 weeks demonstrated higher CASI, MMSE, and IADL scores and achieved a better contrast sensitivity in patients with mild AD when compared to the placebo group, suggesting that EAHE is safe, well-tolerated, and may be important in achieving neurocognitive benefits.

**Clinical Trial Registration:**

ClinicalTrials.gov, identifier NCT04065061.

## Introduction

The pace of population aging across the world over the past half-century is increasing dramatically, triggering a Silver Tsunami of chronic age-related diseases. Among these diseases, Alzheimer’s Disease (AD) is the fifth-leading cause of death among adults aged 65 years and older and is also a leading cause of disability and morbidity (Alzheimer’s Association, 2019). Unlike other major diseases for which there have been steady progress in the development of novel therapies, no new pharmacologic treatment for AD has been approved since 2003 (Hung and Fu, 2017). One theory as to why many intervention trials have failed is that the pathophysiological process of AD is thought to begin many years before the onset of clinical symptoms, and the use of interventions later in the disease may not effectively slow its progression due to established pathological burden (Sperling et al., 2011). As a result, there has been a shift in the clinical research field, with the focus to develop safe and effective interventions in early and presymptomatic AD stages (Graham et al., 2017). To date, several prevention trials have been carried out and shown promising results, suggesting the potential feasibility of implementing non-pharmacological approaches, including dietary interventions (Ngandu et al., 2015; Andrieu et al., 2017).

In a recent study, the lifestyle of 633 Chinese seniors living in Singapore between 2011 and 2017 was analyzed, and it was revealed that various mushrooms have therapeutic effects in combatting AD by exerting neuroprotective and antioxidant effects (Feng et al., 2019). Mushrooms and their extracts have been well-known for their nutritional and culinary values, which may be regarded as novel nature-based nutraceuticals to mitigate AD and other age-related neurodegenerative disorders. In fact, a number of mushrooms including *Hericium erinaceus* (Bull.: Fr.) Pers., *Dictyophora indusiata* (Vent.) Desv., *Grifola frondosa* (Dicks.: Fr.) S.F. Gray, *Tremella fuciformis* Berk, *Tricholoma* sp., *Termitomyces albuminosus* (Berk.) R. Heim, *Lignosus rhinocerotis* (Cooke) Ryvarden, *Cordyceps militaris* (L.:Fr.) Link, *Pleurotus giganteus* (Berk.) Karunarathna and K.D. Hyde, *Ganoderma lucidum* P. Karst, and *Ganoderma neo-japonicum* Imazeki have been reported to have activities related to nerve and brain health (Phan et al., 2017). Among these, the neurohealth properties of *Hericium erinaceus* (Bull.:Fr.) Pers., or its common names Lion’s mane or Monkey’s head mushroom, have been most extensively studied.

Hericenones and erinacines are the two important classes of constitutes isolated from the fruiting body and mycelium of *H. erinaceus*, respectively (Kawagishi et al., 1991, 1992, 1994, 1996a,b; Lee et al., 2000). Both hericenones and erinacines are low-molecular weight, relatively hydrophobic compounds, and proven to stimulate nerve growth factor (NGF) synthesis and promote NGF-induced neurite outgrowth in nerve cells *in vitro* (Lai et al., 2013). However, hericenones failed to promote NGF gene expression in 1321N1 human astrocytoma cells (Mori et al., 2008) while erinacine A successfully upregulated the NGF level in the locus coeruleus and hippocampus of rats (Shimbo et al., 2005). To date, only erinacines A (unpublished results) and S (Hu et al., 2019) but not hericenones have been verified to cross the blood-brain-barrier, suggesting a greater of likelihood of them targeting the central nervous system. Furthermore, the *in vivo* neuroprotection of erinacine A-enriched *H. erinaceus* (EAHE) mycelia has been demonstrated in several studies against stroke, Parkinson’s disease, AD, depression, and aging (Li et al., 2018b). Based on these findings, it is highly suggestive that erinacine A is one of the key components responsible for the neurotrophic and neuroprotective activities of *H. erinaceus*.

A previous human pilot study has been carried out to investigate the efficacy of oral administration of *H. erinaceus* with 50- to 80-year-old Japanese men and women diagnosed with mild cognitive impairment. The subjects in the *H. erinaceus* group took four *H. erinaceus* tablets three times a day for 16 weeks and showed an improvement in cognitive functions (Mori et al., 2009). However, in this study, the active constituents, representative markers, and major chemical constituents of *H. erinaceus* tablets have not been extensively addressed. While there is still a controversy regarding whether hericenones in the *H. erinaceus* fruiting body have neuroprotective activities *in vivo*, erinacine A in the *H. erinaceus* mycelia, on the other hand, confers neuroprotective effects and attenuates the oxidative stress against stroke (Lee et al., 2014), AD (Tzeng et al., 2018), Parkinson’s disease (Kuo et al., 2016), depression (Chiu et al., 2018), and aging (Li et al., 2019) in mouse models. As there is an urgent need to translate basic discovery research to clinical evaluation, this is the first clinical investigation of three *H. erinaceus* mycelia capsules (350 mg/capsule; containing 5 mg/g erinacine A active ingredient) per day for the treatment of patients with early AD.

## Materials and Methods

### Sample Preparation and High-Performance Liquid Chromatography (HPLC) Analysis

*Hericium erinaceus* mycelia enriched with 5 mg/g erinacine A were prepared and evaluated according to a procedure described previously (Li et al., 2014b). In brief, EAHE mycelia was grown in a submerged liquid medium comprised of 0.25 % yeast extract, 4.5% glucose, 0.5% soybean powder, 0.25 % peptone, and 0.05 % MgSO_4_ with an initial pH set to 4.5 at 26°C for 5 days. This process is then scaled up in 500-L and 20-ton fermenters for 5 days and 12 days, respectively. Following mass production, the mycelia were lyophilized, extracted with methanol, and analyzed by HPLC to quantify 5 mg/g erinacine A in EAHE mycelia. For study’s intervention, 350 mg EAHE mycelia were encapsulated in each gelatin capsule and used as treatments.

### Study Design

The present study was a 1-year, double-blind, randomized, placebo-controlled, fixed-dose intervention pilot trial conducted at Chung Shan Medical University in patients with mild AD. The study protocol was approved by the Institutional Review Board of Chung Shan Medical University and registered with ClinicalTrials.gov under the number NCT04065061. This study comprised of a 3-week no-drug screening period, followed by a 49-week double-blind treatment period with 2-parallel groups in which eligible patients were randomized to either three 350 mg/capsules containing 5 mg/g erinacine A per day or identical appearing placebo capsules with meals. This dose was chosen according to a previous study design (Li et al., 2019) and converted to human dose as specified by FDA guidelines (FDA, 2005). Cognitive assessments, ophthalmic examinations, biomarker collection, and neuroimaging were followed throughout the study period. Written informed consent from all patients or their legal representatives was obtained before their enrollment.

### Participants, Randomization, and Blinding

The inclusion criteria for enrollment included patients with age >50 years and diagnosis of probable AD according to the Diagnostic and Statistical Manual of Mental Disorders (fourth edition, DSM-IV) (American Psychiatric Association, 2013) and National Institute of Neurological and Communicative Disorders and Stroke–Alzheimer’s Disease and Related Disorders Association (Mckhann et al., 1984) criteria. The exclusion criteria included patients with severe somatic or psychiatric comorbidity as they may significantly impair cooperation with the study. Once a participant met the study’s eligibility criteria, a baseline visit was planned, and thorough somatic and neurological examinations were carried out.

**FIGURE 1:**
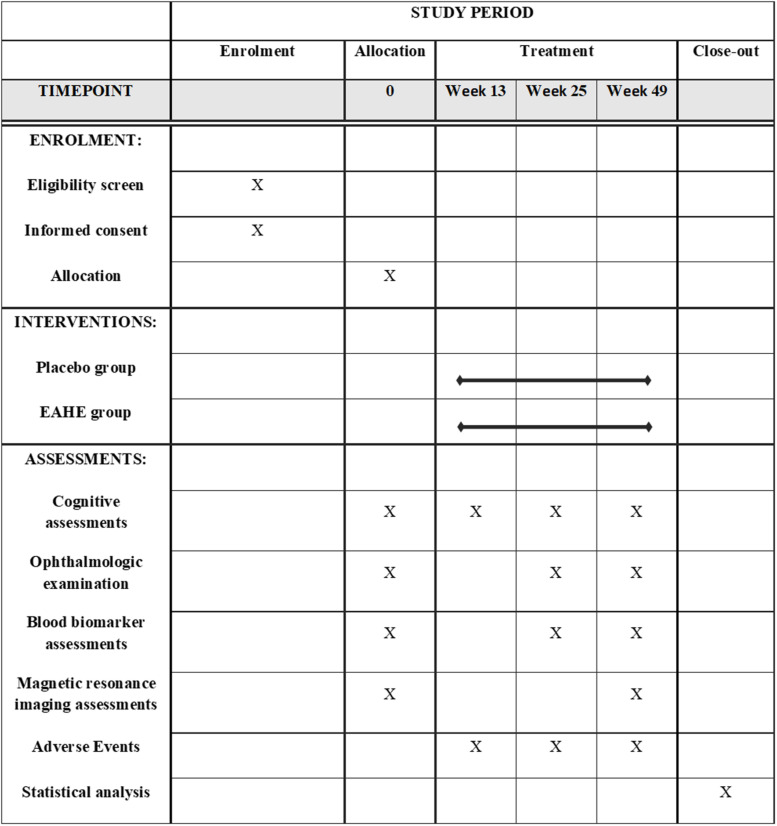
Schedule of enrollment, interventions and assessments (SPIRIT Figure).

Following the baseline assessments, participants were randomly assigned to receive either the placebo or three EAHE mycelia capsules per day according to a randomization list produced by a computerized random-number generator. Except for two trial-independent statisticians that were unmasked, all patients, caregivers, raters, and investigators were blinded to the interventions until the database was finalized. The schedule of trial enrollment, interventions, and assessments according to the Standard Protocol Items: Recommendations for Interventional Trials (SPIRIT) Statement (Chan et al., 2013) is presented in Figure 1.

### Efficacy and Safety Parameters

All participants received either three EAHE mycelia capsules per day or placebo for 49 weeks and were assessed by a rater at 0, 13, 25, and 49 weeks after commencing the treatment. The efficacy of EAHE mycelia was determined by the mean change from baseline to the final analysis and was evaluated by a comprehensive battery, which included cognitive assessments, ophthalmic examinations, biomarker collection, and neuroimaging.

The cognitive assessments were performed at baseline, week 13, week 25, and week 49. Reference measures for cognition included the Neuropsychiatric Inventory (NPI) (Cummings et al., 1994), Cognitive Abilities Screening Instrument (CASI) (Teng et al., 1994), Mini-Mental State Examination (MMSE) (Folstein et al., 1975), and Instrumental Activities of Daily Living (IADL) (Nygard, 2003). These standard tests are used extensively in both clinical practice and research to measure treatment effects in patients with mild to moderate dementia.

A complete ophthalmologic examination including the measurement of best-corrected visual acuity (BCVA) and contrast sensitivity (CS) was conducted at baseline, week 25, and week 49. Monocular and binocular best-corrected distant visual acuity were determined using a standard clinical Snellen eye chart at a 5-meter distance from the chart. The contrast sensitivity test was performed with a standard Pelli-Robson chart under the same conditions for all the patients.

Blood biomarkers were collected at baseline, week 25, and week 49. After overnight fasting, blood samples from each patient were drawn through 22-gauge needles and transferred into either ethylene diamine tetraacetic acid-potassium (EDTA-K2) tubes for hematological analysis or stored in tubes without anti-coagulants for biochemical analysis. For hematological analysis, homocysteine (Hcy) and hemoglobin (Hb) were measured using an automated hematology analyzer (Gen-STM, Beckman Coulter, Inc., United States) while the serum biochemistry parameters including albumin and calcium were performed using an automated biochemistry analyzer (LX^®^-20, Beckman Coulter, Inc., United States). Quantitative determination of other blood biomarkers such as alpha1-antichymotrypsin (α-ACT; ab171574, Abcam, United Kingdom), amyloid-beta peptide 1–40 (β-amyloid; CEA864Hu, Wuhan USCN Business Co., Ltd., China), apolipoprotein E4 (APOE4; K4699, BioVision Inc., United States), dehydroepiandrosterone-sulfate (DHEAS; ab108669, Abcam, United Kingdom), brain-derived neurotrophic factor (BDNF; KA0329, Abnova, Taiwan), and superoxide dismutase (SOD; #19160, Sigma-Aldrich, United States) were measured using commercially available enzyme-linked immunosorbent assay (ELISA) kits.

Neuroimaging such as magnetic resonance imaging (MRI) assessment was performed before and after the intervention period. All subjects had brain imaging using diffusion tensor imaging (DTI) through a Siemens Magnetom Skyra three-tesla (3T) scanner. Diffusion datasets were collected with the following parameters: repetition time (TR) = 4800 ms, echo time (TE) = 97 ms, field of view (FOV) = 25 cm, image matrix = 128 × 128, slice number = 35, thickness = 4 mm, flip angle = 90°, 4 *b*-values = 0, 1000, 1500, 2000 s/mm^2^, diffusion direction = 64, and bandwidth = 1562 Hz/pixel. The fiber number, the fractional anisotropy (FA), and the apparent diffusion coefficient (ADC) from the arcuate fasciculus (ARC), parahippocampal cingulum (PHC), inferior fronto-occipital fasciculus (IFOF), and uncinate fasciculus (UNC) regions in the dominant and non-dominant hemispheres were determined based on the diffusion tensor analyzed through using the specialized software nordicICE v4.0.2.

Safety was evaluated by monitoring adverse events according to the Food and Drug Administration regulations (Behrman Sherman et al., 2011). Moreover, adverse event reporting was also reviewed by an independent safety monitoring committee systematically throughout the study.

### Sample Size and Statistical Analysis

Considering this study is a pilot study to assess the cognitive efficacy of EAHE mycelia in patients with mild AD and the feasibility of a further larger clinical trial, a total of at least 60 people were recruited based on a simulation study to maintain adequate power while keeping the overall sample size of the pilot and main trial together to a minimum (Teare et al., 2014). Statistical analyses were performed using SPSS software (version 18). Data are presented as means ± standard deviation (SD). The Mann–Whitney U test was used to compare the data between the two study groups while the Wilcoxon signed-rank test was used to compare variables before and after the intervention. The statistically significant value was set at *p* < 0.05.

## Results

### Participants

**FIGURE 2:**
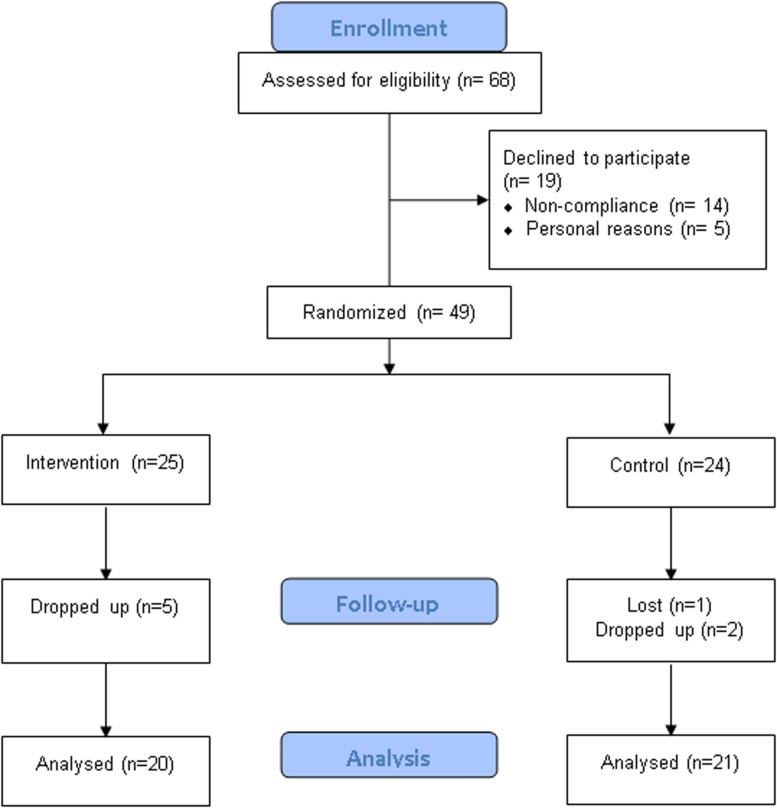
CONSORT diagram.

A total of 68 participants who had been diagnosed with mild AD were recruited in this study. Participants were randomly assigned to either the EAHE intervention group or the placebo control group. Of the 68 patients who participated in the surveillance, 19 declined to participate due to personal reasons (*n* = 5) and non-compliance (*n* = 14). Forty-nine participants were randomized, of whom seven subjects withdrew consent and one subject lost to follow-up. In the end, 41 subjects completed the study, and the data from 17 male and 24 female study participants were analyzed (Figure 2). Demographic and baseline characteristics are shown in Table 1. There were no statistical differences between the EAHE group and the placebo group in gender, age, and education characteristics at baseline.

**TABLE 1:** Participant demographics.

**Variables**	**EAHE group (*n* = 20)**	**Placebo group (*n* = 21)**	***p*-Value**
Gender (male/female)	6/14	11/10	–
Age (years)	74.3 ± 7.15	77.05 ± 8.2	0.261
Education (years)	6.35 ± 4.74	6 ± 5.36	0.826

*Values are given as mean ± SD.*

### Cognitive Assessments

The NPI, CASI, MMSE, and IADL tests were performed at baseline, after weeks 13, weeks 25, and weeks 49 of supplementation with EAHE. The means scores of each parameter in the EAHE and placebo group are presented in Table 2. For the NPI test, the mean NPI scores at all time-points decreased in both the EAHE and placebo groups compared to the baseline. Although subjects who received EAHE has a lower mean NPI score than the placebo group at week 49 (0.67 ± 1.15 vs. 2.25 ± 3.3), the comparison to baseline values showed no significant difference in both groups (*p* = 0.077 and 0.163, respectively). Moreover, when compared to the baseline values and different time points of CASI scores, subjects in the EAHE group showed an increasing trend with marginal significance (71.75 ± 17.12 to 75.35 ± 15.86; *p* = 0.058) whereas the subjects in the placebo group showed a decreasing trend from the baseline to 49 weeks (73.52 ± 14.51 to 69.67 ± 16.62; *p* = 0.064). However, there was no significance in relation to the intra-group and inter-group CASI analysis except for the difference between the baseline and week 25 of the placebo group (*p* = 0.043). Furthermore, MMSE scores significantly increased (21.75 ± 6.1 to 23.2 ± 5.92; *p* = 0.035) in the EAHE group from the baseline to week 49 whereas the comparison of all time-points showed no significant change in the placebo group. Nevertheless, all the pairwise comparisons of the MMSE test were not statistically significant (*p* > 0.05). Finally, for the IADL test, no baseline differences between the EAHE and placebo groups at any time points were observed except for the pairwise comparison at week 49 which was statistically significant (6.7 ± 2.47 vs. 5 ± 2.7; *p* = 0.012, respectively).

**TABLE 2:** Comparison of cognitive assessments between EAHE and placebo groups.

	**EAHE group (*n* = 20)**		**Placebo group (*n* = 21)**		
		
**Variables**	**Value**	**Intragroup *p*-value**	**Value**	**Intragroup *p*-value**	**Intergroup *p*-value**
NPI					
Baseline	4.21 ± 6.62	–	3 ± 5.2	–	0.696
Week 13	0 ± 0	0.306	1.33 ± 1.53	0.812	0.259
Week 25	1.5 ± 2.12	0.439	1.33 ± 2.31	0.087	0.989
Week 49	0.67 ± 1.15	0.077	2.25 ± 3.3	0.163	0.129
CASI					
Baseline	71.75 ± 17.12	–	73.52 ± 14.51	–	0.804
Week 13	72.74 ± 15.83	0.246	70.71 ± 19.3	0.13	0.881
Week 25	73.8 ± 17.15	0.313	69.62 ± 16.07	0.043*	0.368
Week 49	75.35 ± 15.86	0.058	69.67 ± 16.62	0.064	0.315
MMSE					
Baseline	21.75 ± 6.1	–	21.33 ± 5.74	–	0.629
Week 13	22.58 ± 5.6	0.065	20.95 ± 6.47	0.616	0.4
Week 25	22.55 ± 6.24	0.23	21.05 ± 5.5	0.686	0.353
Week 49	23.2 ± 5.92	0.035*	20.67 ± 6.17	0.661	0.261
IADL					
Baseline	6.35 ± 2.81	–	5.71 ± 2.65	–	0.25
Week 13	6.37 ± 2.31	1	6 ± 2.07	0.484	0.423
Week 25	6.45 ± 2.58	0.705	5.57 ± 2.29	0.634	0.11
Week 49	6.7 ± 2.47	0.157	5 ± 2.7	0.075	0.012*

*Values are given as mean ± SD. NPI = Neuropsychiatric Inventory; CASI = Cognitive Abilities Screening Instrument; MMSE = Mini mental state Examination; IADL = Instrumental Activities of Daily Living. ^∗^*p* < 0.05.*

### Ophthalmologic Examination

Table 3 summarizes the ophthalmologic examination for the EAHE and placebo groups after 25 and 49 weeks of intervention. The analysis of BCVA in OD (right eye), OS (left eye), and OU (both eyes) of the EAHE and placebo groups showed no difference from their baselines to the end of the study. Although significant baseline differences in BCVA of OD, OS, and OU were found between the groups, these differences remained unchanged throughout the study period except for BCVA OS at week 25 (*p* = 0.101). Meanwhile, subjects with EAHE treatment showed improvements in the mean monocular CS (OD: 0.84 ± 0.19 to 0.90 ± 0.08; OS: 0.83 ± 0.2 to 0.86 ± 0.13) and binocular CS (OU: 0.88 ± 0.15 to 0.89 ± 0.11) values following 49 weeks of intervention whereas the placebo group showed an upward trend in the CS OD (0.72 ± 0.32 to 0.77 ± 0.27), a downward trend in the CS OS (0.83 ± 0.18 to 0.78 ± 0.17), and remained unchanged (0.85 ± 0.19 to 0.85 ± 0.08) in the CS OU at the end of the study. Nevertheless, all these groups did not reach statistical significance except for the changes in CS OU from baseline to 49 weeks in the placebo group (0.85 ± 0.19 to 0.85 ± 0.08; *p* = 0.033) and differences of CS OS at week 49 between treatment groups (*p* = 0.046).

**TABLE 3:** Comparison of ophthalmologic examination between EAHE and placebo groups.

	**EAHE group (*n* = 20)**		**Placebo group (*n* = 21)**		
**Variables**	**Value**	**Intragroup *p*-value**	**Value**	**Intragroup *p*-value**	**Intergroup *p*-value**
BCVA OD					
Baseline	0.83 ± 0.24	–	0.59 ± 0.33	–	0.013*
Week 25	0.8 ± 0.27	0.552	0.59 ± 0.33	0.814	0.026*
Week 49	0.83 ± 0.2	0.545	0.57 ± 0.28	0.258	0.005*
BCVA OS					
Baseline	0.82 ± 0.25	–	0.63 ± 0.27	–	0.024*
Week 25	0.77 ± 0.27	0.153	0.67 ± 0.23	0.633	0.101
Week 49	0.82 ± 0.24	0.824	0.64 ± 0.23	0.201	0.025*
BCVA OU					
Baseline	0.9 ± 0.22	–	0.75 ± 0.23	–	0.017*
Week 25	0.89 ± 0.26	0.923	0.74 ± 0.21	0.472	0.027*
Week 49	0.88 ± 0.22	0.948	0.69 ± 0.2	0.104	0.005*
CS OD					
Baseline	0.84 ± 0.19	–	0.72 ± 0.32	–	0.249
Week 25	0.86 ± 0.19	0.257	0.75 ± 0.29	0.187	0.212
Week 49	0.9 ± 0.08	0.257	0.77 ± 0.27	0.582	0.089
CS OS					
Baseline	0.83 ± 0.2	–	0.83 ± 0.18	–	0.323
Week 25	0.82 ± 0.25	0.85	0.82 ± 0.11	0.227	0.155
Week 49	0.86 ± 0.13	0.606	0.78 ± 0.17	0.13	0.046*
CS OU					
Baseline	0.88 ± 0.15	–	0.85 ± 0.19	–	0.28
Week 25	0.88 ± 0.2	0.739	0.86 ± 0.09	0.405	0.069
Week 49	0.89 ± 0.11	0.68	0.85 ± 0.08	0.033*	0.056

*Values are given as mean ± SD. BCVA = best-corrected visual acuity; CS = contrast sensitivity; OD = oculus dexter; OS = oculus sinister; OU = oculus uterque. ^∗^*p* < 0.05.*

### Blood Biomarker Assessments

With further analysis of the blood biomarkers over the 49-week study period within the groups (Table 4), significant improvements of Hcy at week 25 and 49 (*p* = 0.007 and *p* = 0.012, respectively) were observed in the EAHE group while significant negative effects in calcium at week 25 (*p* = 0.004), albumin at week 49 (*p* = 0.004), Hb at week 25 and 49 (*p* = 0.003 and *p* = 0.009, respectively), and BDNF at week 25 (*p* = 0.012) were noted in the placebo group. Moreover, although both groups showed significant decreases in SOD and APOE4 as well as significant increases in α-ACT and β-amyloid (*p* < 0.05), APOE4, α-ACT, and β-amyloid had an improving trend in the EAHE group than the placebo group at week 49. No significant difference, however, was observed for all other parameters.

**TABLE 4:** Comparison of blood biomarkers between EAHE and placebo groups.

	**EAHE group (*n* = 20)**		**Placebo group (*n* = 21)**		

**Variables**	**Value**	**Intragroup *p*-value**	**Value**	**Intragroup *p*-value**	**Intergroup *p*-value**
Calcium					
Baseline	9.15 ± 0.29	–	9.23 ± 0.33	–	0.407
Week 25	9.05 ± 0.34	0.406	9.06 ± 0.4	0.004*	0.743
Week 49	9.08 ± 0.2	0.238	9.12 ± 0.47	0.23	0.545
Albumin					
Baseline	4.62 ± 0.29	–	4.35 ± 0.23	–	0.297
Week 25	4.35 ± 0.24	0.336	4.28 ± 0.31	0.387	0.503
Week 49	4.28 ± 0.26	0.657	4.14 ± 0.22	0.004*	0.136
Hb					
Baseline	13.35 ± 1.93	–	13.82 ± 1.55	–	0.251
Week 25	13.04 ± 1.6	0.102	12.73 ± 3.27	0.003*	0.411
Week 49	13.29 ± 1.58	0.822	13.4 ± 1.69	0.009*	0.611
Hcy					
Baseline	11.44 ± 4.52	–	11.39 ± 4.6	–	1
Week 25	9.23 ± 2.96	0.007*	10.11 ± 3.57	0.099	0.449
Week 49	9.42 ± 3.05	0.012*	11.85 ± 6.56	0.794	0.225
SOD					
Baseline	66.94 ± 10.43	–	62.39 ± 11	–	0.411
Week 25	66.63 ± 13.89	0.737	65.52 ± 13.98	0.274	0.917
Week 49	48.39 ± 19.28	0.001*	52.1 ± 17.76	0.021*	0.715
BDNF					
Baseline	16817.78 ± 7269.27	–	14146.35 ± 5248.59	–	0.235
Week 25	14790.69 ± 13578.16	0.117	9725.25 ± 6780.67	0.012*	0.335
Week 49	17943.1 ± 7356.27	0.654	13793.27 ± 5545.83	0.821	0.068
DHEAS					
Baseline	1.45 ± 1	–	1.11 ± 0.99	–	0.211
Week 25	1.45 ± 0.97	0.627	1.08 ± 0.76	0.59	0.211
Week 49	0.91 ± 1.07	0.167	0.87 ± 0.82	0.689	0.465
α-ACT					
Baseline	201499.24 ± 183201.7	–	205721.68 ± 186042.47	–	0.766
Week 25	5438979.8 ± 5500347	0.001*	4130525.7 ± 4307187.3	<0.001*	0.584
Week 49	367337.35 ± 263536.7	0.019*	426465.17 ± 267768.25	0.017*	0.175
APOE4					
Baseline	141.45 ± 137.36	–	144.47 ± 230.67	–	0.167
Week 25	33.52 ± 28.7	0.01*	29.81 ± 29.63	0.073	0.549
Week 49	30.19 ± 35.04	0.001*	20.52 ± 20.01	<0.001*	0.511
β-amyloid					
Baseline	125.47 ± 86.34	–	104.11 ± 73.49	–	0.404
Week 25	156.24 ± 94.38	0.086	124.59 ± 74.04	0.244	0.397
Week 49	313.32 ± 122.45	0.015*	317.77 ± 124.98	0.001*	0.531

Values are given as mean ± SD. Hb = hemoglobin; Hcy = homocysteine; SOD = superoxide dismutase; BDNF = brain-derived neurotrophic factor; DHEAS = dehydroepiandrosterone-sulphate; α-ACT = alpha 1-antichymotrypsin; APOE4 = apolipoprotein E4; β-amyloid = amyloid-beta peptide 1–40. *p < 0.05.

### Magnetic Resonance Imaging (MRI) Assessments

The total fiber number, FA, and ADC values from the ARC, PHC, IFOF, and UNC regions in the dominant and non-dominant hemispheres of the EAHE and control group are listed in Table 5. After 49 weeks of EAHE intervention, the total fibers were significantly less decreased than those in the placebo group. Nevertheless, they did not reach statistical significance between groups (*p* = 0.715). In addition, compared to their baselines, the mean ADC values from the ARC region in the dominant hemisphere significantly increased in the placebo group while the ADC values from the PHC region in the dominant hemisphere significantly decreased in the EAHE group at week 49. No statistically significant differences were found in other parameters.

**TABLE 5:** Comparison of MRI assessments between EAHE and placebo groups.

	**EAHE group (*n* = 20)**		**Placebo group (*n* = 21)**		
**Variables**	**Value**	**Intragroup *p*-value**	**Value**	**Intragroup *p*-value**	**Intergroup *p*-value**
Total fibers					
Baseline	43523 ± 8327.67	–	41463.43 ± 8868.05	–	0.44
Week 49	40085.21 ± 9124.5	0.001*	38512.2 ± 11643.18	0.008*	0.715
D.ARC.FA					
Baseline	0.45 ± 0.03	–	0.43 ± 0.03	–	0.05
Week 49	0.45 ± 0.03	0.421	0.43 ± 0.04	0.205	0.148
N.ARC.FA					
Baseline	0.45 ± 0.03	–	0.45 ± 0.03	–	0.86
Week 49	0.46 ± 0.02	0.144	0.46 ± 0.03	0.469	0.704
D.PHC.FA					
Baseline	0.39 ± 0.02	–	0.4 ± 0.03	–	
Week 49	0.4 ± 0.02	0.609	0.4 ± 0.03	0.756	
N.PHC.FA					
Baseline	0.38 ± 0.03	–	0.36 ± 0.1	–	0.797
Week 49	0.4 ± 0.03	0.266	0.38 ± 0.03	0.861	0.272
D.IFOF.FA					
Baseline	0.47 ± 0.02	–	0.44 ± 0.03	–	0.024*
Week 49	0.47 ± 0.02	0.879	0.44 ± 0.05	0.513	0.014*
N.IFOF.FA					
Baseline	0.46 ± 0.03	–	0.43 ± 0.03	–	0.011*
Week 49	0.46 ± 0.03	0.276	0.43 ± 0.04	0.095	0.006*
D.UNC.FA					
Baseline	0.41 ± 0.03	–	0.4 ± 0.03	–	0.704
Week 49	0.4 ± 0.04	0.463	0.4 ± 0.04	0.828	0.483
N.UNC.FA					
Baseline	0.4 ± 0.03	–	0.4 ± 0.03	–	0.955
Week 49	0.4 ± 0.03	0.421	0.4 ± 0.03	0.962	0.493
D.ARC.ADC					
Baseline	103.63 ± 6.59	–	106.18 ± 7.52	–	0.233
Week 49	104.29 ± 8.84	0.711	109.02 ± 10.63	0.033*	0.105
N.ARC.ADC					
Baseline	103.44 ± 8.65	–	106.66 ± 8.8	–	0.101
Week 49	103.85 ± 11.09	0.372	106.56 ± 10.44	0.365	0.112
D.PHC.ADC					
Baseline	125.51 ± 9.66	–	127.98 ± 12.15	–	0.32
Week 49	123.12 ± 7.68	0.03*	123.82 ± 7.82	0.446	0.8
N.PHC.ADC					
Baseline	126.43 ± 8.8	–	116.56 ± 32.15	–	0.406
Week 49	120.39 ± 3.74	0.05	124.43 ± 7.6	0.6	0.097
D.IFOF.ADC					
Baseline	116.76 ± 7.87	–	121.56 ± 7.97	–	0.17
Week 49	118.02 ± 7.72	0.36	121.82 ± 9.75	0.748	0.226
N.IFOF.ADC					
Baseline	117.71 ± 7.16	–	122.23 ± 9.8	–	0.239
Week 49	118.08 ± 7.23	0.744	120.33 ± 8.19	0.171	0.405
D.UNC.ADC					
Baseline	117.06 ± 3.93	–	119.19 ± 8.82	–	0.579
Week 49	119.57 ± 7.68	0.469	119.06 ± 5.96	0.741	0.849
N.UNC.ADC					
Baseline	117.82 ± 5.57	–	119.48 ± 8.72	–	0.933
Week 49	117.58 ± 5.89	0.307	120.05 ± 7.61	0.276	0.511

Values are given as mean ± SD. D = dominant; N = Non-dominant; ARC = arcuate fasciculus; PHC = parahippocampal cingulum; IFOF = inferior fronto-occipital fasciculus; UNC = uncinate fasciculus; FA = Fractional Anisotropy; ADC = apparent diffusion coefficient. *p < 0.05.

### Adverse Events

During the study, 1 subject lost to follow-up while 7 subjects (7/49; 14.3%) left the study. Reasons for dropout that have been investigated include unsatisfactory efficacy (2 from EAHE group and 1 from the placebo group) and the presence of side effects (3 from EAHE group and 1 from the placebo group). Possible or probable side effects related to the intervention included nausea in the placebo group and abdominal discomfort, nausea, and skin rash in the EAHE group.

## Discussion

Diet is an important modifiable risk factor for AD (Sindi et al., 2018) as it is able to modulate structural brain connectivity (Park et al., 2018), cause positive changes in brain function and behavior (Bolton and Bilbo, 2014), as well as help regulate cognition and emotion (Spencer et al., 2017). As benefits of EAHE associated with brain and nerve health have been well-studied (Li et al., 2018b), this is the first study to endorse its potential in mitigating neurodegenerative disorders. Based on the results of this pilot, randomized, double-blinded, controlled study, subjects with mild AD showed a significant benefit in reducing cognitive decline and improving contrast sensitivity after oral administration of three 5 mg/g EAHE mycelia capsules per day for 49 weeks when compared with placebo.

In this study, through random allocation, the baseline demographic information including age, gender, and education level between EAHE and placebo groups showed no significant differences before the intervention. Nevertheless, after the intervention, a significant deterioration in CASI from baseline to week 25 was noted in the placebo group, a significant improvement in MMSE from baseline to week 49 was observed in the EAHE group, and a significant IADL difference at week 49 were found between the two groups. Higher CASI and MMSE scores represent better cognition, and higher IADL scores represent a lower level of dependence (Chiu et al., 2016). Although there were no significant differences in CASI and MMSE between the EAHE and placebo groups, the scores were higher in the EAHE group compared to those in the placebo group for participants with mild AD, implying that subjects could achieve more benefits from the intervention.

To date, human studies on *H. erinaceus* are scarce. Only three trials were found to examine the efficacy of oral administration of *H. erinacues* for improving brain pathology. In one double-blind placebo-controlled study, 50- to 80-year-old Japanese men and women (*n* = 30) diagnosed with mild cognitive impairment showed marked improvement in cognitive function when compared to controls, using a cognitive function scale based on the revised Hasegawa Dementia Scale and following the effects of oral intake of four 250 mg tablets containing 96% of *H. erinaceus* fruiting body dry powder three times a day for 16 weeks (Mori et al., 2009). In another randomized, double-blind placebo-controlled study, administration of 0.5 g *H. erinaceus* fruiting body in cookies over 4 weeks showed a reduction in anxiety and depression in menopausal women (*n* = 30) compared to those taking placebo, as measured by the Center for Epidemiologic Studies Depression Scale and Indefinite Complaints Index (Nagano et al., 2010). In the third randomized, double-blind, placebo-controlled parallel-group comparative study, the consumption of cookies containing 0.8 g of *H. erinaceus* fruiting body dry powder alleviated the deterioration of short memories and improved the cognitive functions in 31 participants with an average age of 61.3 years old over the period of 12 weeks, as measured by MMSE (Saitsu et al., 2019). Prior studies have reported that NGF could enhance neurogenesis-inducing effects, which led to antidepressant and antianxiety activities (Shohayeb et al., 2018). Although hericenones C and D from the fruiting body of *H. erinaceus* have shown to induce neuroprotective properties (Kawagishi et al., 1991) in rats by stimulating NGF synthesis via activation of the c-jun N-terminal kinase (JNK) pathway, they failed to promote NGF gene expression in 1321N1 human astrocytoma cells (Mori et al., 2008). This result suggested that *H. erinaceus* fruiting body may contains other active compounds and/or hericenones that can potentially improve mild cognitive impairment as well as reduce depression and anxiety.

On the contrary, erinacines isolated from the mycelium of the mushroom are able to pass through the brain-blood barrier into the brain (Hu et al., 2019) to promote NGF synthesis *in vivo* (Shimbo et al., 2005). During normal physiological conditions, NGF is released by the postsynaptic cortical and hippocampal neurons to activate further signaling cascades that include cell survival, maintenance, and proliferation (Biane et al., 2014). However, NGF has been found to be reduced during the pathological conditions of AD, resulting in induced loss of cortical synapses and atrophy of cholinergic neurons in the basal forebrain (Iulita and Cuello, 2014). Moreover, analyzing AD11 anti-NGF transgenic mice that express NGF antibodies in the brain, it was observed that NGF deprivation leads to early inflammation and Alzheimer’s neurodegeneration (Capsoni et al., 2011). In this regard, as erinacine A has been proven to promote NGF synthesis *in vivo*, it may contribute to the survival and regeneration of cholinergic neurons as well as revive cholinergic signaling in the cortex and hippocampus, thereby improving the cognitive ability in subjects with mild AD. However, the precise mechanism of its action needs further investigation.

Not only could NGF markedly protect degenerating neurons in the brain, studies have also shown that NGF administration could modulate the development and differentiation of the retina and the optic nerve, as well as promote the survival and recovery of retinal ganglion cells (Aloe et al., 2012). To our knowledge, this is the first study to examine the efficacy of EAHE on visual acuity and contrast sensitivity. No significant differences were observed in the ophthalmologic examination in this study except for a higher CS OS at week 49 after EAHE treatment. This finding of EAHE as a NGF stimulator in improving CS but not VA in subjects with mild AD is consistent with previous studies. They have found that contrast sensitivity was significantly reduced in patients with AD compared to elderly control subjects while no significant difference in visual acuity were found between the patients with AD and control subjects (Crow et al., 2003) suggesting that EAHE targeting astrocytes only responded to an injury or damaged area by maintaining neurogenesis as a mechanism of repair (Poulose et al., 2017). Future studies, however, are required to further explore this possible mechanism.

With EAHE consumption, it is important to note that an altered diet or a multiplicity of environmental changes could change the blood proteome as well as ions (Te Pas et al., 2013). In different studies, EAHE treatment was accompanied by improvements in blood biomarkers in subjects with mild AD. Biomarkers monitoring based on biochemical analysis of blood during or after the intervention period could offer considerable promise for improving the treatment of AD (Cummings et al., 2019). Recent studies have identified a biomarker panel that included blood-based markers that significantly increased alpha-1-Antichymotrypsin, β-amyloid, superoxide dismutase, and homocysteine levels as well as decreased calcium, albumin, dehydroepiandrosterone sulfate, apolipoprotein E, hemoglobin, and BDNF levels in AD (Dekosky et al., 2003; Laske et al., 2011; Doecke et al., 2012; Ng et al., 2019; Pan et al., 2019). Consistent with the current study, biomarkers such as calcium, albumin, APOE4, Hb, and BDNF were significantly lowered while α-ACT and β- amyloid were significantly elevated during the study period in the placebo group. However, there were no significant changes in calcium, albumin, Hb, and BDNF compared to the baseline and a trend toward improving SOD, APOE4, and α-ACT levels were observed in the EAHE group, indicating that EAHE may have possible effects in arresting or delaying further neurodegenerative processes.

The effects of EAHE on the rate of neurodegeneration could also be detected using advanced MRI, such as DTI, to probe human brain microstructures (Cho et al., 2008). DTI provides quantitative measures of FA and ADC, which enable the assessment of the cellular microstructure and fiber tract integrity in live tissues. Fiber tracts such as PHC and UNC contribute to learning and memory, and IFOF and AF contribute to language functioning (Mcdonald et al., 2008). These were evaluated in each subject to depict their global white matter status. In this study, although a statistical significance was not found between the groups, the total fibers of six fiber tracts calculated were significantly less decreased than those in the placebo group after EAHE intervention, suggesting that EAHE ameliorates the loss of fiber numbers by stimulating NGF synthesis and inducing neurogenesis. Moreover, within the six fiber tracts, the mean ADC values from the ARC region in the dominant hemisphere significantly increased in the placebo group while no significant difference was found in the EAHE group in comparison to their baselines, implying that there was a greater disorganization in the neural structure observed in the placebo group. Moreover, ADC values from the PHC region in the dominant hemisphere significantly decreased in the EAHE group whereas no significant difference was found in the placebo group when compared to their baselines, indicating that there was a more well-organized neural structure noted in the EAHE group. These results tie well with a previous study wherein the ADC values increased in the ARC and PHC of patients with mild AD when compared with a control group (Mayo et al., 2019), signifying that EAHE could improve structural deterioration of ARC and PHC in patients with mild AD.

Lastly, despite four subjects who dropped out during the study period due to reported adverse events such as abdominal discomfort, nausea, and skin rash, no other adverse event were reported. The overall incidence was 8.2% during the entire 49-weeks. However, due to the increasing trend in clinical practice to treat elderly patients with multiple medications (Poleksic and Xie, 2019), it remains a challenge to identify if these adverse events were caused by EAHE consumption. Yet, reports on genotoxicity (Li et al., 2014a), acute toxicity (Li et al., 2018a), 28 days subchronic toxicity (Li et al., 2014b) 90 days subchronic toxicity (Lee et al., 2019) and teratotoxicity (Li et al., 2018a) have been conducted in animals and showed no adverse effects. Moreover, no adverse events have been reported after the launch of EAHE products into the Taiwanese market since 2015 (Li et al., 2018b). Nevertheless, further studies, especially the serum biochemical and hematological data along with urinalysis values after long-term consumption in humans, are important to consider.

In comparison to the placebo group, the intake of EAHE for 49 weeks showed higher CASI, MMSE, and IADL scores and achieved a better contrast sensitivity in patients with mild AD. The benefit of EAHE in reducing cognitive decline may be associated with improved blood biomarkers such as calcium, albumin, Hb, Hcy, SOD, BDNF, APOE4, and α-ACT, as well as reduced structural deterioration in the ARC and PHC regions of patients with mild AD. However, further studies on the mechanism of action of EAHE at the biochemical and molecular levels are necessary. In addition, although EAHE is safe and well-tolerated, a larger study is required to determine the benefits of EAHE consumption for patients with MCI or mild AD.

## Data Availability Statement

All datasets generated for this study are included in the article/supplementary material.

## Ethics Statement

The study protocol was approved by the Institutional Review Board of the Chung Shan Medical University and registered with ClinicalTrials.gov under the number NCT04065061. The patients/participants provided their written informed consent to participate in this study.

## Author Contributions

I-CL analyzed the data and wrote the manuscript. H-HC, C-HL, and T-HL conceived and performed the experiments. W-PC, L-YL, Y-WC, and Y-PC provided the reagents. C-CC and DP-CL provided the expertise and feedback.

## Conflict of Interest

Grape King Bio Inc., provided support in the form of salaries for the authors I-CL, W-PC, L-YL, Y-WC, Y-PC, and research materials, but did not have any additional role in the study design, data collection and analysis, decision to publish, or preparation of the manuscript. The remaining authors declare that the research was conducted in the absence of any commercial or financial relationships that could be construed as a potential conflict of interest.
